# Diagnosis of Incipient Phthisis

**Published:** 1885-09-20

**Authors:** E. Van Goidtsnoven

**Affiliations:** Atlanta, Georgia


### Southern Medical Record.

EDITORS:

THOMAS S. POWELL, M. D. R. C. WORD, M. D.

*R. C. WORD, M.D., Managing Editor.*

☞All Communications and Letters on Business connected with the RECORD must be addressed to the Managing Editor.

VOL. XV. ATLANTA, GA., SEPTEMBER 20, 1885. No. 9.

### ORIGINAL AND SELECTED ARTICLES.

### DIAGNOSIS OF INCIPIENT PHTHISIS.

BY E. VAN GOIDTSNOVEN, M. D., ATLANTA, GEORGIA.

Read before the Atlanta Society of Medicine, August, 1885.

I will not offend your intelligence by insisting upon the importance of the diagnosis of incipient phthisis, and of all the facts which lead to the early recognition of the disease. We are all aware of the insidiousness of phthisis; and it rarely occurs that any of us are called upon to treat the disease *ab initio.* It would be, to say the least, the height of presumption on my part to attempt laying down any rules whereby an exact diagnosis, made at the commencement of the disease, could be subsequently verified by a *post mortem* examination. The differentiation so nicely laid down in the books seems inapplicable to many of the cases, because of the complications present at a given time, while, in some one instance, the character of disease at different periods has seemed to fairly represent separate varieties. "All diseases," says Dr. James Henry Bennett, "are greatly modified in their symptoms and progress, as also in the results of the treatment to which they are subjected, by the form or type which they assume, from the first development; and none more so than pulmonary consumption. On the above grounds," says he, "I prefer to study these types on a chemical basis, rather than on an obscure histological one ; that is, I prefer to refer the types of the disease to general pathology, which best explains them. At the bedside" (I still quote from Dr. Bennett), "I have tried in vain to discriminate between catarrhal, pneumonic, scrofulous, fibroid phthisis, whereas the laws of pathology have proved a safe guide." How, then, it may be asked, can confidence be placed in data pointing out a stage the forerunner of so many succeeding conditions, or extending, in many cases, over so long a period of time ?

Not to instruct, but rather to invite discussion, with an earnest hope to arrive at the elucidation of many nebulous points which cloud my knowledge on this subject, have I selected this theme : I simply search for information.

In the course of my investigations, I chanced, lately, to read the clinical lectures of Professor Grancher, of Paris. They present new ideas of the highest interest to the medical reader ; and, although the phenomena in question are susceptible only of delicate interpretation, and are not easily accessible to proper valuation, yet, owing to their practical utility, I have endeavored to summarize them as briefly as seems consistent with the proper conception of their agency, and concluded to lay them here before you for consideration. It should be remarked, *a priori*, that, in a clinical point of view, the respiratory act presents three phenomena, closely connected with each other, constituting, as it were, a physiological association, and manifesting themselves through physical signs easily elicited by auscultation, percussion, and palpitation. Let one of these signs undergo some modification, and we at once witness an unquestionable pathological disssociation, which, when accompanied by general phenomena, suffice, in many cases, to diagnose phthisis in the affirmative sense.

Amongst these pathological dissociations, the most important one, with a view of reaching this conclusion, is that in which respiration presents some alterations, although neither the sound of percussion nor the vibrations in palpitation are in the least modified ; and in this study of dissociated respirations particular attention must be paid to the modification of inspiration, which are of far greater value than those of expiration.

The modification which the *quality* of the respiratory murmur is liable to undergo should be first considered. It constitutes that phenomenon which has been designated by many authors as rude, bronchial, cavernous, amphoric, etc. *It is observed especially under the clavicle*, and is of greatest importance as a sign of incipient phthisis, especially when its *duration* seems to be permanent and resists all forms of treatment. Moreover, whenever the inspiration, rude in quality, is *lowered* in tone, it is of very grave import. This fact has hitherto escaped the attention of diagnosticians, and Professor Grancher calls the special attention of the profession to this point as clearly characterizing the alteration in question.

This chain of thoughts and association of ideas with respect to pathological respiration have also been entertained and advocated by Professor Prat, as can be seen from his pointing out an interesting fact demonstrating that the question of *tone* in the respiratory murmur had also engaged his attention. For, this observer states, that inspiration and expiration bear the same musical relation to each other as that which exists between the superior and inferior plates of a violin—the former, made of pine, gives the *re* or *b;* the latter, made of maple, gives the *do* or *a*—the difference being that of a full tone. This remark applies to the normal condition only ; and here ends all further remarks by the author on this subject. Yet, in all pathological cases in question, there is no longer any difference between the two stages of respiration ; both give the same note, the *tone* of inspiration being lowered. This particularity, easily recognized, enables the diagnostician to properly authenticate that variety of respiration wherein inspiration alone is modified, it being then not only rude but lowered in tone.

The respiratory act may also be modified in its duration. The inspiration is then shorter, and one may also observe in this case a phenomenon to which the name of Disproportioned Respiration would be applicable. It consists in this fact, that, notwithstanding the great capacity for action of the respiratory movement, the quantity of air which penetrates the lungs is much less considerable than the movement would seem to indicate. It should also be stated that, in nearly every instance wherein inspiration is short it is more or less rude.

With the briefness of inspiration, weakness not unfrequently coincides. This last modification is of less import than the preceding ones, because it often manifests itself at a more advanced stage of the disease, when all other signs have already made act of presence. It also indicates deeper and more developed lesions ; yet this grave significance should not always be adhered to as a conclusive inference, as it may be due to pleuro-pneumonic adhesions without any lesion of parenchyma.

The *rhythm*, when altered, produces an interrupted or jerked respiration, which has been variously interpreted by different authors—some attaching to it a very grave character ; others giving it but a secondary importance. This alteration may be met in both periods, but is much more frequent in inspiration than in expiration. The inspiration is then ordinarily effected in three periods, the respiratory murmur being now soft, then rude. This characteristic, under such conditions, may be considered momentous, but its import is considerably lessened by the fact that it is very difficult to discriminate it from the interrupted respirations which are observed in other morbid conditions of a very different character; for they can be either of cardiac origin or the consequence of nervous lesion. Hence, the reason why, in the minds of many diagnosticians, the alterations of expiration have far less value than those of inspiration.

The dissociated respirations, the principal type of which consists in the rudeness of inspiration (percussion, and palpitation, eliciting no appreciable lesion), give us, therefore, sufficient signs and proofs of pulmonary tuberculosis, provided, however, that they be observed at one apex only—that this condition be permanent and accompanied by certain rational concomitants capable of sustaining the suspicions which such conditions are likely to evoke.

The following illustration will show under what conditions tuberculosis may be diagnosed :

Here is a girl, nineteen years of age ; she never had any cough, but her menstruation is irregular and laborious ; she is anæmic ; somewhat out of health, and presents herself to the hospital, complaining of lassitude only. Upon auscultation, a short and weak respiration is detected on the right side, but rude and low on the left under the clavicle ; posteriorly it is ruder still. This rudeness is of such accentuation as to enable the diagnostitician to ascertain that the apex respiration appears as full as that of the middle lobe, which is contrary to a physiological or normal condition. The patient complains of no trouble about the chest ; yet her looks betray, in some way, a tuberculous diathesis, and further examination discloses antecedents of grave import on the part of her mother. Although these physical signs are so little accentuated as would escape examination, were not the latter very close and attentive, Professor Grancher does not hesitate to diagnose this case incipient tuberculosis.

In order to fix a proper value on such delicate shades of auscultation, Professor Grancher suggests the following *modus operandi:* Let the mind of the diagnostician disconnect the two periods of respiration ; let it first listen only to the inspiration in the various points where its study is deemed necessary ; let it then listen to the expiration. It should be borne in mind that expiration is rarely altered without the inspiration having first undergone some modification in a greater or lesser degree. With a little practice, the ear can travel over various portions of the thoracic region during the period of expiration, and thus hear only the inspiration, the murmurs of which can be compared with each other and differentiated as the auscultation moves on over these parts. In a normal or physiological condition, the inspiration is unvariable in its duration, tone, and quality, whatever may be the point of auscultation. On the contrary, it varies considerably in its intensity according to the points explored. The maximum of intensity may be found in the subclavian and axillary regions ; its minimum in the supraspinous fossa. It may be said, in a general way, that with regard to the posterior portion, this intensity goes on increasing from the upper to the lower thoracic limits ; in the process of expiration the very opposite phenomenon is observed, its intensity increasing from the lower to the upper limits. It should be added, that as far as expiration is concerned, its physiological estimates are far more variable than those of inspiration, which considerably lessens its import in the summing up of all the reliable material necessary to form a perfect diagnosis.

The manifestations revealed by percussion are harder of interpretation than those presented by auscultation. Professor Grancher has drawn from the sign of subclavian tympanism some very precise indications of immense utility in the diagnosis of pleurisy, of its amount and nature, and most especially in its prognosis.

In tuberculosis, percussion, as regards diagnosis in the first period, is but secondary, and only serves to confirm the data of auscultation. Nevertheless, in order to value it aright, it is necessary to be fully conversant with the results it produces in a physiological condition. In this juncture, special attention is called to a new fact. All authors on the subject admit that the result given by percussion of the lungs in a normal state, is not a sound but a murmur, which it would be impossible to render by any note, whilst tympanism has all the character of a musical sound.

Now, it has been of late ascertained—and this is a fact of physiological interest—that in a normal condition the percussion sound gives out a note nearly fixed in all adults, in men and women alike, and in the same region. This sound would correspond to the *do* or *a* of the fourth octave, in the middle and lower regions, posteriorly as well as anteriorly. In the upper parts the sound is more acute, and one obtains the *do* or *a* of the fifth octave in percussing the clavicular, infra-clavicular regions, and the supra-spinous fossa.

Palpation may, in some cases where it is impossible to discover alterations in thoracic vibrations, produce very useful results for the diagnosis of incipient phthisis ; but in order to interpret them properly, one should be thoroughly acquainted with certain physiological particularities. We know, for instance, that vibrations are in direct proportion to the *intensity* of the voice, its quality, and the thickness of the thoracic parietes. In women, vibrations are almost null ; yet—and this is a fact which has had thus far no practical application—vibrations invariably missing at the base may be detected in the superior portion of the lungs. With man, on the contrary, the maximum of vibrations is found at the base, if he have a basso voice ; over the middle portion, if the tone of his voice be materially higher. Moreover, if, whilst exploring the vibrations in a man having a basso voice, one causes him to speak in a falsetto or head tone, it will be ascertained that the maximum of vibrations, which manifested itself at the base of the lung, changes its locality, and transfers itself to the superior regions in proportion as the voice raises its tone. These variations of vocal vibrations in their relation with the tone of the voice present, therefore, a series of delicate shades, the knowledge of which may be of immense utility in many embarrassing cases of diagnostic investigation.

All authors are unanimous in recognizing that tuberculosis is first made manifest at the apices of the lungs. Which of the two apices is first affected is a question which has not been as easily determined, since Laennec and other observers after him maintain that it is the right, whilst Andral and Louis contend that it is the left. Recent researches on this point would tend to demonstrate that the first stage of tuberculosis begins with the right apex in subjects engaged in manual labor, in either worn out or alcoholic patients, and in nearly all cases of acquired tuberculosis. On the other hand, tuberculosis would make its *debut* on the left apex in patients of sedentary habits, or hitherto engaged in pursuits requiring but little activity. In women, this location is also generally first observed. But whatever may be the opinions of authors on this point, it is a matter of wonder that none of them has thus far paid any attention to the phenomena of auscultation, which are conjointly presented at the base of the lung during that first period, phenomena which are plainly detected in all cases of incipient tuberculosis. Auscultation, in this first stage, will, in effect, reveal the fact that, whilst alterations are detected at the apex, another focus lies at the base, presenting nearly the same symptomatology. Here, indeed, respiration is equally modified, especially in the inspiratory process, which is rude, low, and somewhat snoring. This can be ascertained by comparing the base of the affected side with that of the sound one. It even often occurs that this differentiation is better established between the bases than between the apices; and Professor Grancher remarks that he not unfrequently obtains better and more satisfactory results from the signs of the lower regions of the lung, when those of the apices do not respond with all the clearness that is usually obtained. At any rate, this is a reliable supporting point in the process of diagnosis which should not be neglected or discarded as a mere pathological curiosity. This is not the only sign presented by auscultation at the base of the lung, for, not unfrequently, a certain degree of dry pleurisy is manifested in the immediate region of the seventh or eighth rib, through some friction accompanying this particular modification of the inspiration which has just been mentioned. It is, moreover, a remarkable fact that those lesions remain confined at the base whilst, at the same time, the intermediate region of the lung presents not the slightest sign of alteration.

Lastly, we may hear also in the same region some mucous rales, somewhat variable, and produced at the end of the inspiration; they are fine subcrepitant rales, indicating a certain degree of pulmonary congestion. Phenomena of this kind have been already noticed by other diagnosticians, who, however, account for them in a different way, and attribute them to the temporary compression of the pneumo-gastric nerve.

One may, therefore, find in this early period of phthisis, at the base of the lung, auscultatory phenomena under three different forms, which can be either grouped or isolated, and are the more remarkable as they are generally unaccompanied by any symptoms, such as cough, pain or dyspnœa. It must be added, moreover, that reference is here made only to accessory phenomena, having no direct relation to the presence of tubercules in the points where the latter are met. The evolution of these lesions of the base in the first stage can, in every particular, be likened to that of the upper lesions; for, in the slow forms of tuberculosis, when one apex is invaded after another, its corresponding base is invaded also; so that a true parallelism and pathological solidarity unite the base, as well as the apices. Hence, at a more advanced period of the disease the lesion of the bases is arrested in its evolution probably by the supplementary respiration which then takes place owing to the increasing impermeability of the upper portion of the lung.

Such is the ordinary progress of the slow form of tuberculosis, which invariably tends to become bilateral; but there are cases where tuberculosis remains absolutely unilateral. Professor Jaccoud has called special attention to these latter facts, and shown that sudden, brusk, and well accentuated tuberculosis generally assumes this form. To this may be added certain forms of hemorragic tuberculosis, and some cases of a pleuritic character, with great effusion. Grancher claims for these views such a correctness as to very frequently enable him, without interrogating the patient, but simply determining the typography of the lesion, to qualify the form of evolution of the disease, *slow* if the lesion be bilateral, *rapid*, on the contrary, if it be unilateral.

This last observation, however, is applicable only to the first phasis of the tuberculous evolution, for, at a more advanced stage of the disease, it usually occurs that both lungs are involved by tuberculosis, which, for a long time, had confined its ravages to but one.

